# Sanguinarine synergistically potentiates aminoglycoside‐mediated bacterial killing

**DOI:** 10.1111/1751-7915.14017

**Published:** 2022-03-23

**Authors:** Chang Lu, Nian Zhang, Sihoi Kou, Liangliang Gao, Bo Peng, Yunlu Dai, Jun Zheng

**Affiliations:** ^1^ 59193 Faculty of Health Sciences University of Macau Macau China; ^2^ School of Life Sciences Sun Yat‐sen University Guangzhou 510006 China; ^3^ 474988 Laboratory for Marine Biology and Biotechnology Qingdao National Laboratory for Marine Science and Technology Qingdao 266071 China; ^4^ 59193 Institute of Translational Medicine University of Macau Macau China

## Abstract

Aminoglycosides are one of the oldest classes of antimicrobials that are being used in current clinical practice, especially on multi‐drug resistant Gram‐negative pathogenic bacteria. However, the serious side effects at high dosage such as ototoxicity, neuropathy and nephrotoxicity limit their applications in clinical practice. Approaches that potentiate aminoglycoside killing could lower down their effective concentrations to a non‐toxic dosage for clinical treatment. In this research, we screened a compound library and identified sanguinarine that acts synergistically with various aminoglycosides. By checkerboard and dynamical killing assay, we found that sanguinarine effectively potentiated aminoglycoside killing on diverse bacterial pathogens, including *Escherichia coli, Acinetobacter baumannii, Klebsiella pneumonia* and *Pseudomonas aeruginosa*. The mechanistic studies showed an elevated intracellular ROS and DNA oxidative level in the bacterial cells treated by a combination of sanguinarine with aminoglycosides. Furthermore, an enhanced level of sanguinarine was observed in bacteria in the presence of aminoglycosides, suggesting that aminoglycosides promote the uptake of sanguinarine. Importantly, sanguinarine was shown to promote the elimination of persister cells and established biofilm cells both *in vivo* and *in vitro*. Our study provides a novel insight for approaches to lower down the clinical dosages of aminoglycosides.

## Graphical abstract

Aminoglycosides primarily interact with the bacterial ribosomes and result in protein misfolding and ROS production. Insertion of abnormal proteins into the cell membrane increases the membrane permeability and leads to enhanced uptake of sanguinarine. The sanguinarine acts synergistically with aminoglycoside to generate high level of ROS and results in bacterial death.

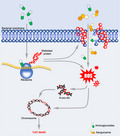

## Introduction

The rapid emergence and wide dissemination of antibiotic resistant pathogens, as well as the declining antibiotic discovery pipelines, have made antibiotic resistance become a global crisis. Multidrug resistance (MDR) among the ‘ESKAPE’ pathogens (Rice, 2008) – namely *Enterococcus faecium*, *Staphylococcus aureus*, *Klebsiella pneumonia*, *Acinetobacter baumannii*, *Pseudomonas aeruginosa* and *Enterobacter* species – especially constitutes the most threats to our lives. These pathogens have acquired resistance to most, if not all, antibiotics that are currently available in the clinic (Pendleton *et al*., 2013; De Oliveira *et al*., 2020). Unfortunately, the endeavour to combat resistance by discovering new antibiotics has become uniquely difficult. On one hand, bacteria have evolved an effective barrier of cell envelope to prevent unwanted compounds from entering the cells (Lomovskaya and Lewis, 1992; Li *et al*., 2015). On the other hand, the low‐hanging fruit, the compounds of natural products, from the various species of actinomycetes, the main source of antibiotics, have been overmined. The pressing crisis of antibiotic resistance and the shrinking novel antibiotic development pipeline call for novel strategies. The revival of old antibiotics emerged to be a new strategy to mitigate the current crisis of antibiotic resistance (Cassir *et al*., 2014).

Aminoglycosides, including kanamycin and streptomycin, represent one of the oldest classes of antimicrobials that are being used in current clinical practice, especially on multidrug resistant Gram‐negative pathogenic bacteria (Pagkalis *et al*., 2011). The advantage of using aminoglycosides is that they retain good activity to many multidrug resistant bacteria, such as *Acinetobacter spp*. and *P. aeruginosa*. However, the clinical usage of aminoglycosides has been limited due to their nephrotoxicity and ototoxicity at higher dosages (Mingeot‐Leclercq and Tulkens, 1999; Wu *et al*., 2002), which can induce extended cortical necrosis and overt renal dysfunction (Parker *et al*., 1982) as well as permanent hearing loss or balance disorders (Pagkalis *et al*., 2011). We have previously shown that mutation in KsgA or modulation on the intracellular concentration of Ap4A could effectively potentiate aminoglycoside killing (Zou *et al*., 2018; Ji *et al*., 2019). These proof‐of‐concept studies promote us to search for chemical compounds that potentiate aminoglycoside killing on bacteria. Such compounds might lower these antibiotics to a non‐toxic dosage for clinical treatment of multidrug resistant infections.

Aminoglycosides are targeting protein synthesis through tRNA mismatching and producing aberrant proteins, eventually resulting in bactericidal outcomes for the cells (Davis *et al*., 1986; Davis, 1987). Mistranslated proteins produced by aminoglycosides ultimately trigger the formation of reactive oxygen species (ROS), especially hydroxyl radicals, that can further oxidize proteins and promote bacterial killing (Kohanski *et al*., 2007, 2008; Dwyer *et al*., 2014). Elevation in the production of hydroxyl radical has been successfully exploited by the genetic engineering approach to enhance the killing efficacy of various groups of antibiotics (Lu and Collins, 2009; Brynildsen *et al*., 2013). These pilot studies clearly demonstrated that enhancing endogenous microbial ROS production is applicable as an approach to potentiate antibiotic activities.

In this study, we screened a compound library containing 693 compounds and identified one that acts synergistically with various aminoglycosides. The identified compound, sanguinarine, was shown to effectively potentiate aminoglycoside killing on diverse bacterial pathogens, including *Escherichia coli*, *A. baumannii*, *K. pneumonia* and *P. aeruginosa*. Further study demonstrated a synergistic effect between aminoglycoside and sanguinarine: aminoglycosides promote the uptake of sanguinarine by bacteria, and sanguinarine in turn enhances aminoglycoside killing. Moreover, the combination of sanguinarine and aminoglycoside resulted in the elevated intracellular ROS and DNA oxidative level. Importantly, we showed that sanguinarine effectively promotes the eradication of bacterial persisters by aminoglycosides both *in vitro* and in mouse infection models. Our study provides a novel insight for approaches to enhance the antibacterial activities of aminoglycoside antibiotics.

## Results

### Identification of compounds potentiating kanamycin inhibition on bacterial growth

**Fig. 1:**
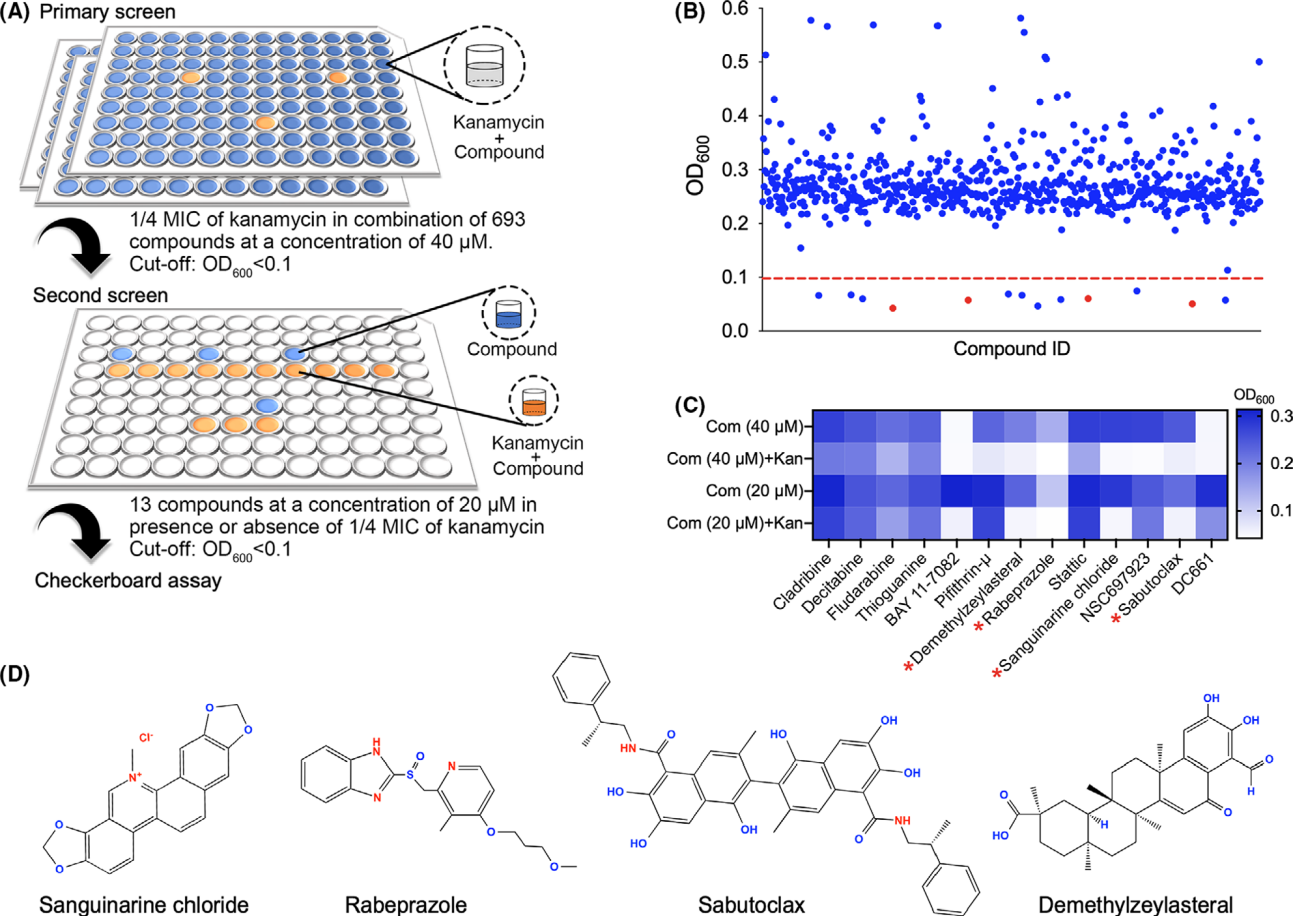
Identification of compounds that potentiate the antibacterial activity of kanamycin. A. Workflow for screening the compound library with *E*. *coli* MG1655. The primary screening was performed at a concentration of 40 μM individual compound with 1/4 MIC of kanamycin. The second screening is performed with the compound at a concentration of 20 μM compound plus 1/4 MIC of kanamycin. B. Dot plot diagram representing the entire screening data used to identify compounds that potentiate kanamycin antibacterial activity. The red dash line delineates the cut‐off for the identification of targets that result in bacterial growth with OD_600_ < 0.1. Dots in red represents the hits from the second screening: demethylzeylasteral, rabeprazole, sabutoclax and sanguinarine chloride. C. OD_600_ value of *E*. *coli* MG1655 after treatment with the 13 primary hits at concentrations of 20 μM in presence and absence of 1/4 MIC kanamycin. D. Molecular structure of sanguinarine chloride, rabeprazole, sabutoclax and demethylzeylasteral. Com, compound; Kan, kanamycin.

To identify compounds that could potentiate aminoglycoside activity, we screened a chemical compound library containing 693 drugs and searched for compounds that enhance the inhibition activities of kanamycin. Bacterial growth was measured in a 96‐well plate with M9 medium supplemented with 1/4 minimum inhibitory concentration (MIC) of kanamycin and 40 μM compounds. An arbitrary cut‐off of OD_600_ = 0.1 was set for the screening (Fig. 1A). A total of 13 compounds was identified at the first screening that potentiated kanamycin inhibition on bacterial growth (Fig. 1B). A second screening on the identified 13 compounds with the combination of 1/4 MIC of kanamycin and individual compound at 20 μM identified four candidates, namely, sanguinarine, rabeprazole, sabutoclax and demethylzeylasterial (Fig. 1C). The structures of four candidates were displayed in Fig. 1D. Importantly, applying these four compounds alone at 20 μM to *E*. *coli* MG1655 did not significantly inhibit bacterial growth (Fig. 1C). Sanguinarine is a plant‐derived benzophenanthridine alkaloid that has previously been demonstrated with various pharmacological activities, such as antimicrobial, anti‐inflammatory and anti‐cancer activities (Kuttikrishnan *et al*., 2019; Fu *et al*., 2021). Rabeprazole is a proton pump inhibitor and the combination of rabeprazole with antibiotics has been used to treat infection by *Helicobacter pylori* in clinical practices (Choi *et al*., 2018). Sabutoclax and demethylzeylasterial are both anticancer compounds that are effective for several resistant cancers (Nguyen *et al*., 2007; Quinn *et al*., 2015; Zhao *et al*., 2017; Hu *et al*., 2018; Zhang *et al*., 2018).

### Sanguinarine promotes the bacterial killing of aminoglycosides

**Fig. 2:**
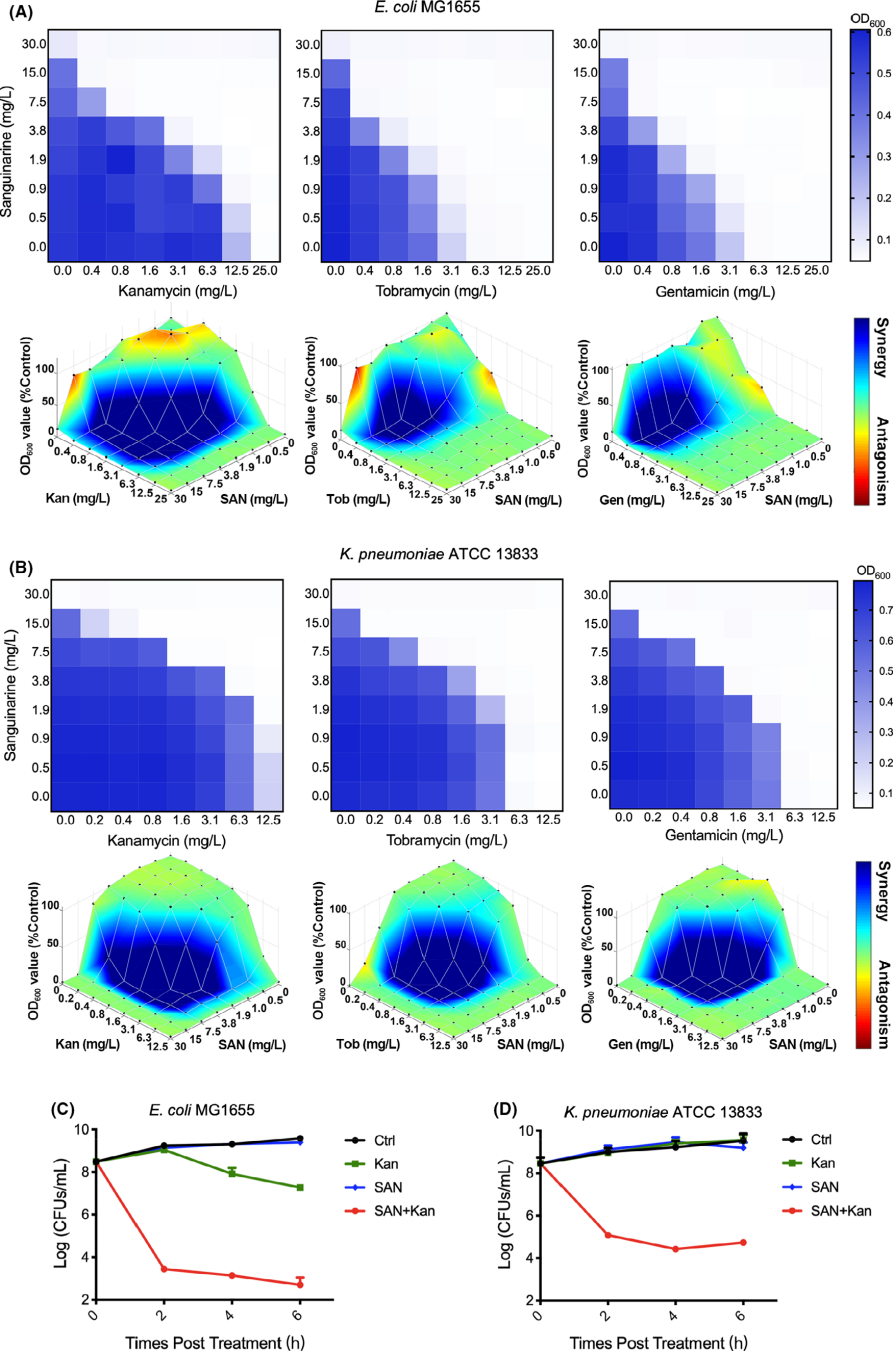
Sanguinarine potentiates the antibacterial activities of different aminoglycosides. A. Checkerboard plots for the antibacterial activity of sanguinarine in combination with selected aminoglycosides on *E*. *coli* MG1655 (top) and the corresponding synergistic effect analysed by Bliss independence criterion was shown (bottom). B. Checkerboard assay (top) and synergy analysis by Bliss independence criterion (bottom) in *K*. *pneumoniae* ATCC 13833. C and D. Sanguinarine potentiates the bacterial killing activity of kanamycin. The bacterial killing activity of kanamycin was tested with *E*. *coli* MG1655 (C), and *K*. *pneumoniae* ATCC 13833 (D) with or without sanguinarine. 1×MIC aminoglycosides and 7.5 mg l^−1^ sanguinarine were used for this experiment. The killing assay was performed at least three times in triplicate. Data are representative of the three independent experiments. The results represent means ± SD (*n* = 3). Ctrl, control; Gen, gentamicin; Kan, kanamycin; SAN, sanguinarine; Tob, tobramycin.

Sanguinarine was chosen to further test whether it is potentiation on kanamycin inhibition that exhibits dose‐dependent synergistic effect. The combination of sanguinarine and various aminoglycosides, including kanamycin, tobramycin, gentamicin, neomycin, amikacin and streptomycin, on bacterial growth was investigated by checkerboard assay with *E*. *coli* MG1655. The drug synergy or antagonism was determined with the most commonly used models, Bliss independence criterion and Loewe additivity model, utilizing Combenefit software (Di Veroli *et al*., 2016; Kinsey *et al*., 2019). Our results showed that the combination of sanguinarine with various aminoglycosides consistently resulted in significantly synergistic effect (Figs 2A, S1A and S2A). Similar results were also observed on *K. pneumoniae* (Figs 2B and S2B). In contrast, no significant synergistic effect was observed between sanguinarine with either β‐lactam antibiotics ampicillin or fluoroquinolone antibiotic norfloxacin (Figs S1B and S2A). Additionally, the synergistic inhibiting effect on the bacterial growth of sanguinarine with kanamycin was not observed on Gram‐positive bacteria (Figs S1C and S2C).

To demonstrate whether the synergistic inhibition effect observed on sanguinarine could be translated into the potentiation on aminoglycoside killing, we examined the killing dynamics of kanamycin in the presence of sanguinarine. Exponential phase *E*. *coli* MG1655 were treated with a combination of 1×MIC of kanamycin and 20 μM (7.5 mg l^−1^) sanguinarine. As shown in Fig. 2C, treatment of bacteria with kanamycin or sanguinarine alone has little effect on the survival of exponential phase bacteria. However, sanguinarine efficiently potentiated the killing activity of kanamycin and a 3‐log decrease in the bacterial survivors was observed in bacteria treated by the combination of sanguinarine with kanamycin compared with those by sanguinarine or kanamycin alone at 1 h post the treatment. Furthermore, the number of bacterial survivors was 6‐log lower in cells treated by kanamycin in the presence of sanguinarine than by kanamycin alone. The potentiation of sanguinarine on bacterial killing in *E*. *coli* MG1655 applies not only to kanamycin but also to other aminoglycosides, including tobramycin, gentamicin and streptomycin (Fig. S3A). Similar potentiation on kanamycin killing was observed in pathogenic bacteria *K*. *pneumoniae* ATCC 13833 (Fig. 2D), *A*. *baumannii* ATCC 17978 and *P*. *aeruginosa* PAO1 (Fig. S3B and C). Our results together suggested that sanguinarine could enhance the bacterial killing activity of various aminoglycosides.

### The combinational use of sanguinarine and aminoglycoside boosts ROS production

**Fig. 3:**
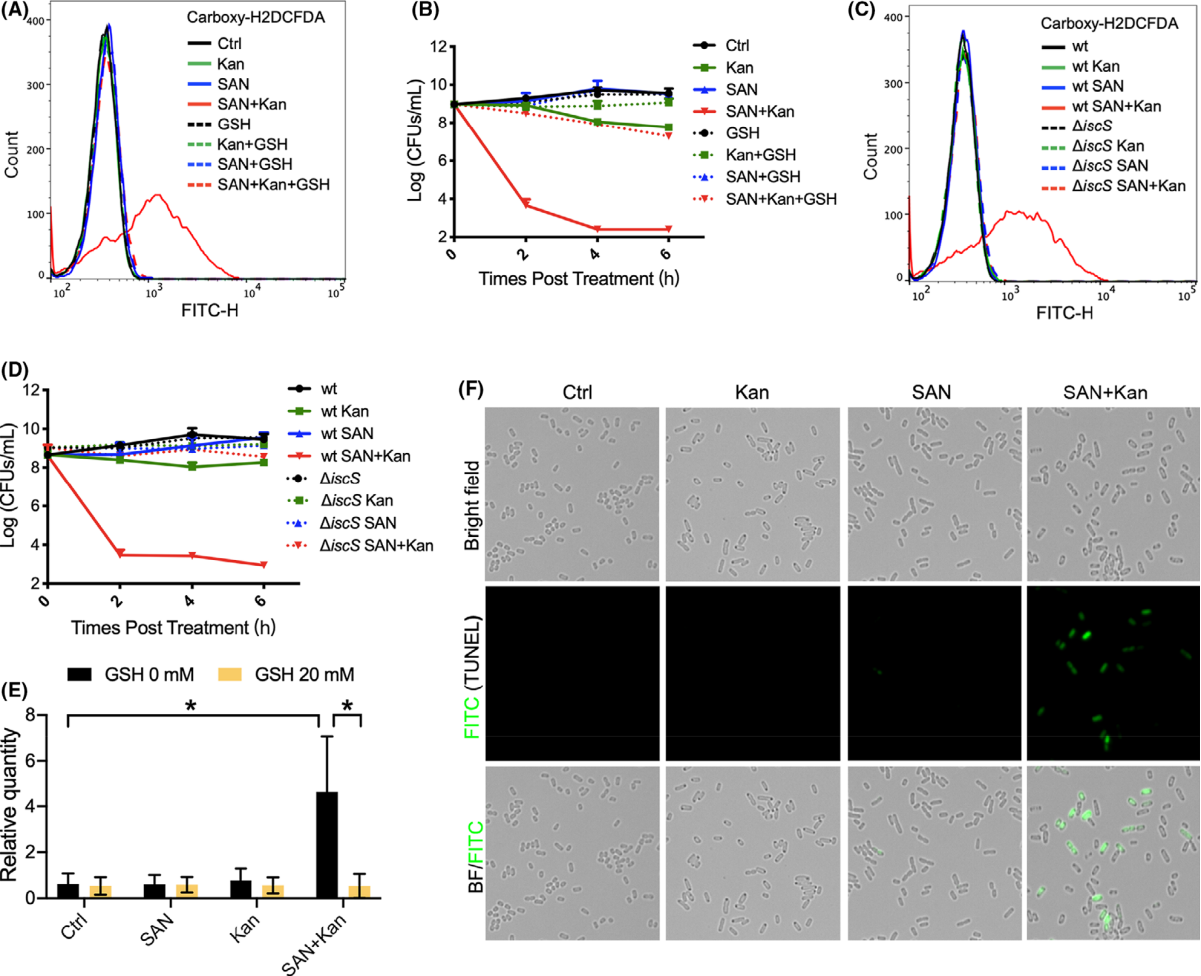
The combination of sanguinarine and aminoglycoside boosts ROS production and DNA oxidative damage. The addition of sanguinarine to bacterial culture treated by kanamycin triggered ROS production (A), which correlates with bacterial death (B). Deletion of *iscS* abolished the production of ROS (C) and bacterial death (D). (E) 8‐oxo‐dG in *E*. *coli* MG1655 was detected by ELISA after 3 h treatment with 1×MIC kanamycin, 7.5 mg l^−1^ sanguinarine, or their combinations in the presence or absence of 20 mM GSH. Quantity of 8‐oxo‐dG was expressed relative to the negative control group (Ctrl). Data are presented as mean ± SD (*n* = 3). Statistical analysis was performed using unpaired *t* test. *P < 0.05; **P < 0.01; ***P < 0.001. (F) TUNEL positive cells in *E*. *coli* MG1655 were observed by fluorescence microscope after 3 h treatment with 1×MIC of kanamycin, 7.5mg l^−1^ sanguinarine alone, or their combinations. Ctrl, control; GSH, glutathione; Kan, kanamycin; SAN, sanguinarine.

The major bacterial killing mechanism of aminoglycoside is to cause protein mistranslation in bacteria, resulting in protein misfolding and aggregation of aberrant proteins (Dobson, 2003). These protein aggregates can further trap other proteins that have essential functions, collectively jeopardizing cell viability (Yang and Hu, 2016). Furthermore, aggregated protein can ultimately trigger the formation of ROS, which causes further oxidative damage on DNA, membranes and proteins (Kohanski *et al*., 2007, 2008; Dwyer *et al*., 2014). Here we found that the ROS level in *E*. *coli* MG1655 treated by kanamycin was significantly enhanced in the presence of sanguinarine as examined by flow cytometry with the ROS‐sensitive fluorescent dye carboxy‐H2DCFDA or hydroxyphenyl fluorescein (HPF) (Figs 3A and S4B). Similarly, a higher intracellular concentration of ROS was also observed in *K*. *pneumonia* ATCC 13833 when treated by a combination of sanguinarine and kanamycin (Fig. S4A).

We next examined whether ROS production played a crucial role in the bacterial killing during the treatment by the combination of aminoglycoside and sanguinarine. With our tested concentration, kanamycin or sanguinarine alone generated a neglectable concentration of ROS in bacterial *E*. *coli*. However, a dramatic increase in ROS concentration was observed in bacteria treated by the combination of kanamycin and sanguinarine. This increase was completely abolished upon the addition of antioxidant glutathione (GSH) (Figs 3A and S4B). Correlated with the diminishment of intracellular ROS concentration, sanguinarine lost the potentiation of the bacterial killing on kanamycin (Fig. 3B). We further constructed an in‐frame deletion mutant of Δ*iscS* in *E*. *coli* MG1655. IscS is essential for the synthesis of the Fe‐S cluster which is involved in the antibiotic‐mediated ROS production (Kohanski *et al*., 2007). Deficiency in *iscS* gene abolished the production of ROS in the presence of sanguinarine (Figs 3C and S4C), and no potentiation of kanamycin killing was observed any longer (Fig. 3D).

Elevated intracellular ROS could damage the nucleotide pool (Foti *et al*., 2012). It was shown that oxidation of the guanine nucleotide pool occurred in bacteria after the treatment by bactericidal antibiotics and the concentration of 8‐oxo‐deoxyguanosine (8‐oxo‐dG) was elevated. 8‐oxo‐dG could then be incorporated into DNA, eventually resulting in double‐strand breaks (DSBs) (Foti *et al*., 2012). We examined the oxidation of guanine in the bacteria treated by the combination of sanguinarine and kanamycin. We found a significantly higher level of 8‐oxo‐dG in bacteria treated by the combination of sanguinarine and kanamycin. In contrast, bacteria treated with either sanguinarine or kanamycin alone at our concentration did not generate a significantly increased level of 8‐oxo‐dG compared to untreated control (Fig. 3E). To further show the correlation of the elevated oxidation of guanine nucleotide pool with the DNA damage, we used terminal deoxynucleotide transferase dUTP nick end‐labelling (TUNEL) assay to examine the fragmentation of bacterial DNA. Our results showed that a significant portion of bacteria treated by a combination of sanguinarine and kanamycin exhibited TUNEL positive. In contrast, a neglectable portion of cells positive in TUNEL was observed in bacteria treated by either sanguinarine or kanamycin alone (Fig. 3F). We further found that neutralization of ROS by GSH abolished the accumulation of 8‐oxo‐dG (Fig. 3E). These results indicate that combining sanguinarine with kanamycin induced severe oxidative damage on bacteria DNA.

### Aminoglycosides promote the uptake of sanguinarine

**Fig. 4:**
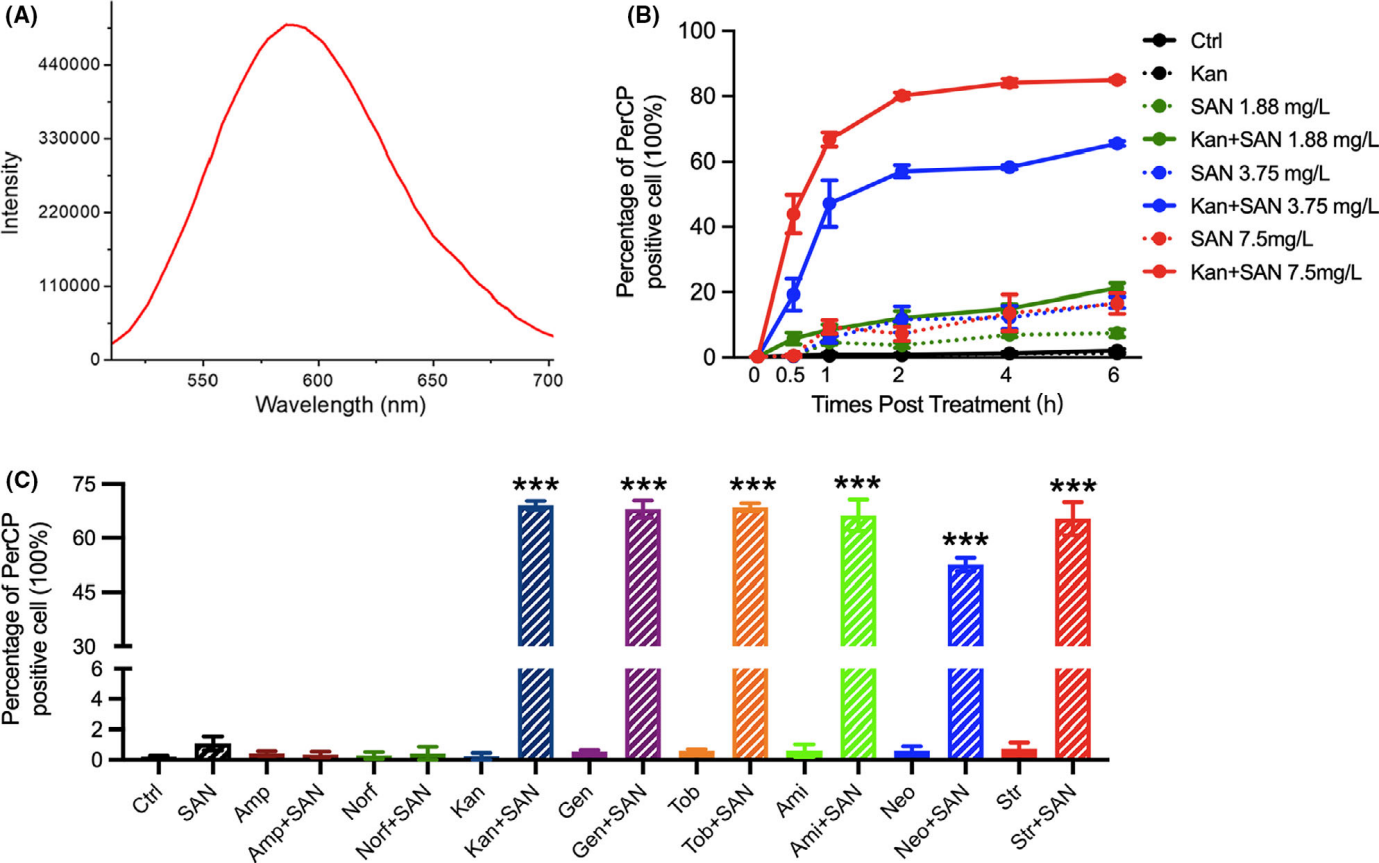
Kanamycin increases the uptake of sanguinarine. A. The emission spectrum of sanguinarine under the 488 nm exciting light. B. Dynamic uptake of sanguinarine in *E*. *coli* MG1655 treated by 1×MIC of kanamycin combinated with or without various concentrations of sanguinarine was determined with PerCP channel by flow cytometry. C. Sanguinarine uptake in *E*. *coli* MG1655 at 1 h post‐treatment with 2×MIC of ampicillin, norfloxacin or 1×MIC of aminoglycosides in the presence or absence of 7.5 mg l^−1^ sanguinarine. Ami, amikacin; Amp, ampicillin; Ctrl, control; Gen, gentamicin; Kan, kanamycin; Neo, neomycin; Nor, norfloxacin; SAN, sanguinarine; Str. streptomycin; Tob, tobramycin. Data are presented as mean ± SD (*n* = 3). Statistical analysis was performed using un‐paired *t* test. *P < 0.05; **P < 0.01; ***P < 0.001.

We serendipitously noticed that sanguinarine could give fluorescence upon excitation. The fluorescence spectrum analysis found that sanguinarine has an emission peak at around 600 nm upon the excitation by 488 nm laser, which correlates well with the characteristics of the PerCP channel of flow cytometry (laser 488 mm, Filter 690/50 nm) (Fig. 4A). Bacterial cells treated by sanguinarine in the presence of aminoglycoside demonstrated a significant level of fluorescence when analysed by the PerCP channel with flow cytometry. We interpreted these fluorescence in the bacteria as the accumulation of sanguinarine inside the cells. We thus monitored the uptake of sanguinarine in bacteria by flow cytometry during the treatment. Exponential phase *E*. *coli* MG1655 was treated by sanguinarine alone or by a combination of sanguinarine with kanamycin. Bacteria were collected at 30 min post treatment and were then subjected to flow cytometry analysis. To our surprise, we found that while the combination of 3.75 mg l^−1^ sanguinarine with 1×MIC kanamycin resulted in a mass population of bacterial cells with strong fluorescence, the bacteria treated by sanguinarine alone barely produced such cells (Fig. 4B). We further investigated the kinetic changes of intracellular fluorescence (which represent intracellular sanguinarine concentration) in bacterial cells treated with three different concentrations of sanguinarine (e.g. 1.88, 3.73 and 7.5 mg l^−1^) in the presence of 1×MIC kanamycin with flow cytometry. Our results showed that the number of bacteria with fluorescence at PerCP started to increase in the bacterial group treated by sanguinarine alone after 30 min and reached plateau, with a very low ratio, approximately at around 2 h post treatment, suggesting that sanguinarine could enter and accumulate in bacterial cells treated by sanguinarine alone at very low efficacy. Enhancement of sanguinare dose for bacterial treatment from 1.88 mg ml^−1^ to 3.75 slightly elevated its intracellular accumulation. However, further increase in sanguinarine dose upto 7.5 mg ml^−1^ did not further alter the accumulation of sanguinarine inside the cells. In sharp contrast, the combination of kanamycin with three concentrations of sanguinarine all resulted in dramatical increase in the number of bacterial cells with fluorescence at PerCP channel with flow cytometry compared to bacteria treated by sanguinarine alone at the respective concentration (Fig. 4B). Importantly, our results demonstrated that the uptake of sanguinarine could also be promoted by other aminolgycosides, such as gentamicin, tobramycin, amikacin, neomyxin and streptomycin in *E*. *coli* MG1655, but not by ampicillin or norfloxacin (Fig. 4C). Thus our results indicate that aminoglycosides, but not β‐lactam or fluoroquinolone, could promote the uptake of sanguinarine by bacteria.

### Sanguinarine potentiates the eradication of biofilm *in vitro*

**Fig. 5:**
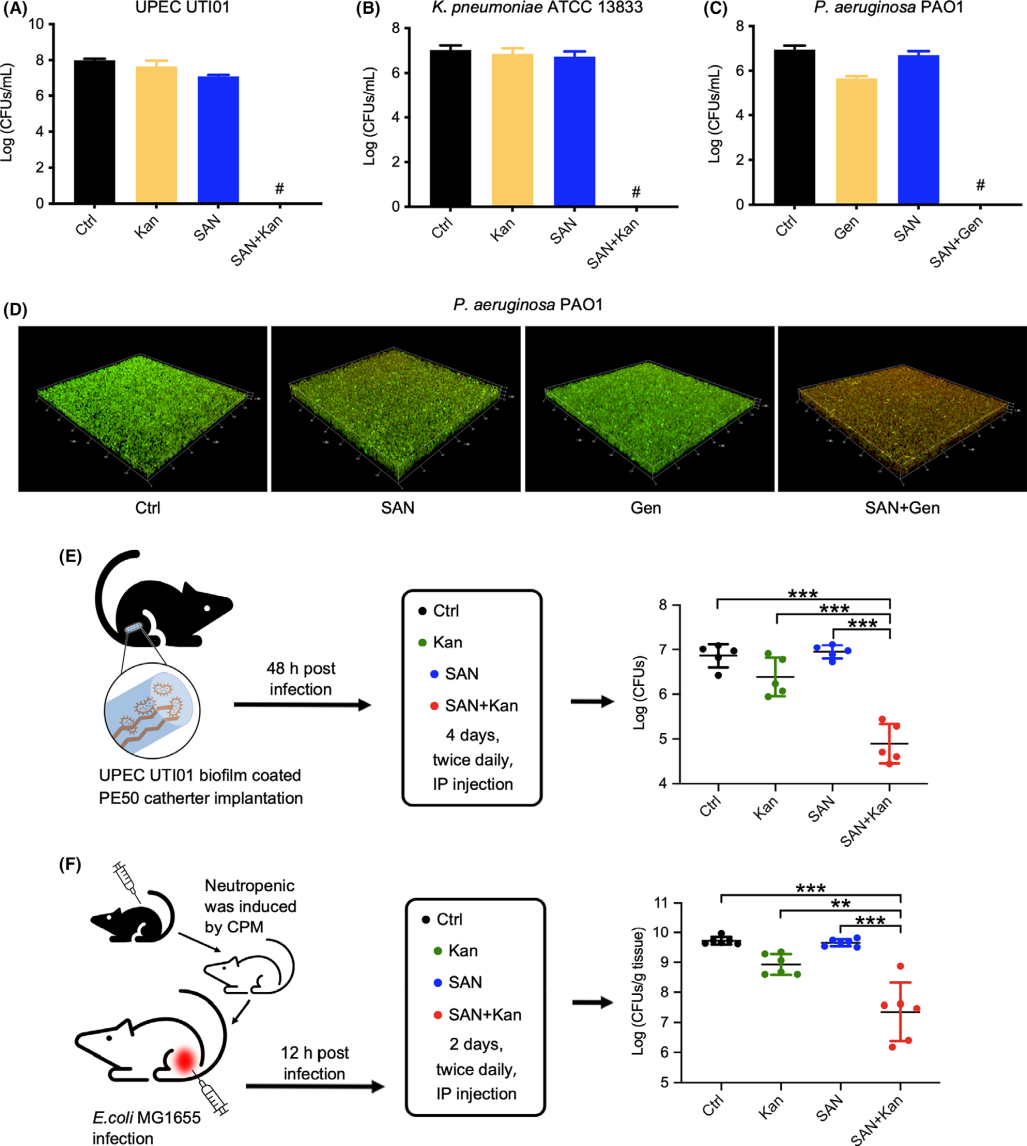
Sanguinarine promotes the eradication of bacteria in biofilm by aminoglycosides. (A–C) Sanguinarine promoted the eradication of bacteria in biofilm formed by UPEC UTI01 (A), *K*. *pneumoniae* ATCC 13833 (B) and *P*. *aeruginosa* PAO1 (C) by kanamycin or gentamicin. ‘^#^’ represents that the CFUs are under the limit of detection. (D) CLSM images of *P*. *aeruginosa* PAO1 biofilms at 6 h post treatment by 1×MIC of gentamicin, 5 mg l^−1^ sanguinarine or their combinations. Biofilms are stained with SYTO9 (green, live) and PI (red, dead). (E) Survival of UPEC UTI01 biofilms on urinary tract‐inserted catheters after treatment with saline (as control), 3 mg kg^−1^ kanamycin, 3 mg kg^−1^ sanguinarine alone, or 3 mg kg^−1^ kanamycin plus 3 mg kg^−1^ sanguinarine (*n* = 5). (F) Survival of *E*. *coli* in neutropenic mice in the infected thigh after treatment with saline, 3 mg kg^−1^ kanamycin, 3 mg kg^−1^ sanguinarine alone, or 3 mg kg^−1^ kanamycin plus 3 mg kg^−1^ sanguinarine (*n* = 6). Ctrl, control; Gen, gentamicin; Kan, kanamycin; SAN, sanguinarine. Data are presented as mean ± SD. Statistical analysis was performed using unpaired *t* test. *P < 0.05; **P < 0.01; ***P < 0.001.

In addition to antibiotic resistance, antibiotic persistence is another major cause of antibiotic treatment failure and relapse of bacterial infections (Lewis, 2010). Importantly, antibiotic persistence promotes the development of antibiotic resistance (Levin and Rozen, 2006; Levin‐Reisman *et al*., 2017; Liu *et al*., 2020). The existence of persister cells is believed to be one of the major reasons behind the recalcitrance of biofilms (Costerton *et al*., 1999; Yan and Bassler, 2019). Sanguinarine has demonstrated strong potentiation on bacterial killing by kanamycin, we next examined whether sanguinarine could help kanamycin to eradicate bacterial persisters. We grew biofilms of a clinical isolated Uropathogenic *E*. *coli* UTI01 (UPEC UTI01) and treated them for 6 h with kanamycin, sanguinarine alone, or the combination of kanamycin with sanguinarine. Sanguinarine or kanamycin alone has little effect on reducing persisters in biofilm. In contrast, the combination of sanguinarine and kanamycin completely eradicated bacteria in the biofilms at 6 h post‐treatment (Fig. 5A). Significant reduction of bacterial survivors was also observed for the treatment of biofilm formed by *K*. *pneumonia* ATCC 13833 (Fig. 5B) and *P*. *aeruginosa* PAO1 (Fig. 5C), and a complete eradication was achieved at 24 h post‐treatment.

We further investigated the combination of sanguinarine with kanamycin on the eradication of biofilm by confocal laser scanning microscopy (CLSM). Bacterial biofilms were treated with drugs for 6 h and the cells were stained by two fluorophores SYTO9 and propidium iodide (PI). Both dyes are able to bind to nucleic acids. While PI penetrates dead cells with disrupted membranes only and displays red colour, SYTO9 enters both live and dead cells and displays a green colour. As shown in Fig. 5D, gentamicin alone at 5 mg l^−1^ did not result in the death of bacteria residing in the biofilm. Sanguinarine has only minor antibacterial activity and only a small portion of the bacteria stained red. In contrast, the combination of sanguinarine with gentamicin kills almost all bacteria and the majority of the cells are stained red, indicating the death of the bacteria (Fig. 5D).

### Sanguinarine promotes aminoglycoside activity in mouse infection models

To demonstrate that sanguinarine could potentiate the eradication of kanamycin on biofilm infection *in vivo* in an infectious mouse model, we examined the biofilm clearance by the combination of sanguinarine with kanamycin in a biofilm‐associated mouse infection model (Allison *et al*., 2011). PE50 catheter contains biofilms of UPEC UTI01 that were implanted in the urinary tract of the female mice. Mice were then treated by kanamycin, sanguinarine alone or their combinations. At 24 h post the last treatment, catheters were collected, the viability of biofilm cells in the catheter was determined. Our results showed that sanguinarine or kanamycin alone has little effect on biofilm clearance. In contrast, the combination kanamycin sanguinarine resulted in more than 100‐fold lower in the number of bacteria recovered (Fig. 5E).

We also examined the therapeutic effect of sanguinarine in a mouse thigh biofilm infection model (Conlon *et al*., 2013). A load of 10^6^ *E*. *coli* was injected into the left thigh of the neutropenic mouse. At 12 h post‐infection, mice were treated with a low concentration of kanamycin, sanguinarine or their combination. At 12 h after the last therapy, thigh muscles were collected and homogenized to count the viable bacteria. We found that kanamycin alone has only a marginal effect on reducing the bacterial load and sanguinarine alone had a negligible effect on bacterial survival. However, the combination treatment by sanguinarine and kanamycin dramatically reduced the bacterial load, which resulted in about 100‐fold fewer bacteria in the mice thigh tissue compared to non‐treated control (Fig. 5F). Taken together, our results suggest that sanguinarine could potentiate the eradication of bacterial persisters by aminoglycosides.

## Discussion

Antimicrobial resistance has been escalating to be a global crisis. As the discovery of novel antibiotics has proven to be uniquely difficult, the revival of the previously abandoned antibiotics becomes an emerging strategy. As one of the oldest classes of antibiotics, aminoglycosides retained good activity to drug‐ or multidrug‐resistant bacteria. However, its toxicity has discouraged the wide use of these groups of antibiotics in the clinic. Previously, the proof‐of‐concept studies demonstrated that targeting other pathways or enhancing ROS could sensitize these groups of antibiotics to a potentially lower, non‐toxic concentration (Lu and Collins, 2009; Brynildsen *et al*., 2013; Zou *et al*., 2018; Ji *et al*., 2019). In this study, we screened a compound library and identified sanguinarine that acts synergistically with various aminoglycosides. We showed that sanguinarine effectively potentiated aminoglycoside killing on diverse bacterial pathogens. In addition, sanguinarine promoted the eradication of bacterial biofilm and persisters infection by aminoglycosides both *in vitro* and in the mouse infection models.

Similar to infection by drug‐resistant bacteria, biofilm‐associated infections, such as urinary tract infections, chronic wound infections, infections of indwelling devices or cystic fibrosis lung infection are notoriously recalcitrant to antibiotic treatment (Lebeaux *et al*., 2014). The drug tolerance of biofilms at least partially attributes to persisters as the main component of biofilm (Lewis, 2001; Spoering and Lewis, 2001; Yan and Bassler, 2019). The inefficiency of antibiotic eradication of bacteria in biofilm is one major cause of antibiotic treatment failure and relapse of bacterial infections (Lewis, 2010). Importantly, bacteria tolerant to antibiotic killing is also the fertile ground for the development of resistance that promotes its occurrence of resistance (Levin and Rozen, 2006; Levin‐Reisman *et al*., 2017; Windels *et al*., 2019; Liu *et al*., 2020). The potentiation of aminoglycosides by sanguinarine not only enhanced the killing of fast‐growing bacteria but also resulted in the quick eradication of persisters in the biofilm. This observation suggested that the sanguinarine can not only reduce the clinical concentration of aminoglycoside but also potentially reduce the rate of emergence of aminoglycoside resistant mutation. These results provide a new insight to revive aminoglycosides in the future.

**Fig. 6:**
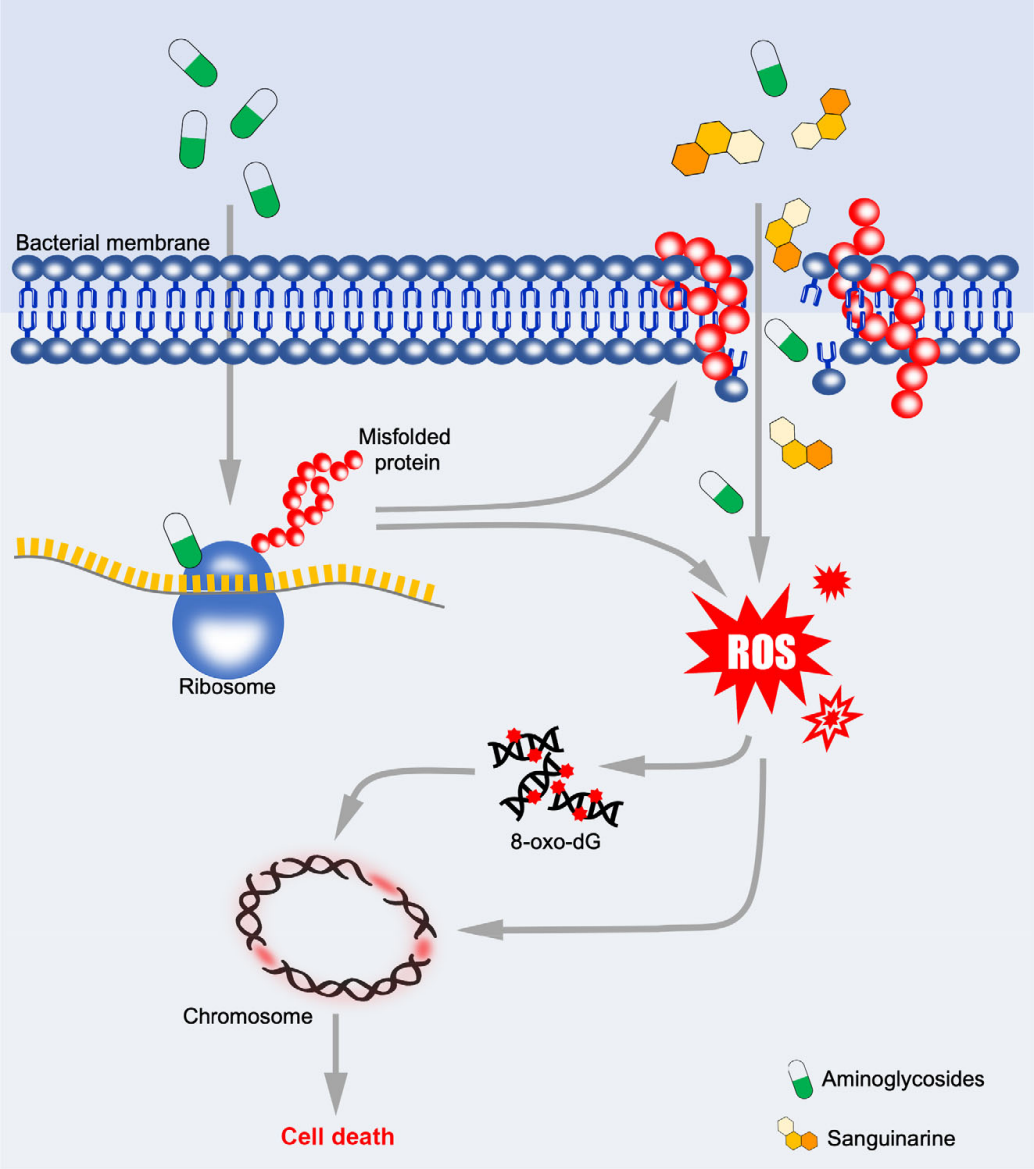
Proposed model for potentiating mechanism of sanguinarine on aminoglycosides. Aminoglycosides primarily interact with the bacterial ribosomes and result in protein misfolding and ROS production. Insertion of abnormal proteins into the cell membrane increases the membrane permeability and leads to enhanced uptake of sanguinarine. The sanguinarine acts synergistically with aminoglycoside to generate high level of ROS and result in bacterial death.

Sanguinarine is a plant‐derived benzophenanthridine alkaloid with a wide spectrum of pharmacological activities (Croaker *et al*., 2016). Sanguinarine alone was found to bear anti‐bacterial activity, likely through inhibiting FtsZ and disturbing the septa formation (Beuria *et al*., 2005; Obiang‐Obounou *et al*., 2011). It also demonstrates good anti‐biofilm activity in *Serratia marcescens* and fungi *Candida albicans* (Zhong *et al*., 2017; Fu *et al*., 2021). However, our current results showed that sanguinarine alone has weak anti‐bacterial and anti‐biofilm activity at the physiological concentration on a broad range of bacteria cultured *in vitro*. Instead, we showed that when sanguinarine is combined with aminoglycosides, it efficiently potentiated the antibacterial killing activity of aminoglycoside antibiotics and enabled the eradication of biofilm. This synergistic effect of aminoglycosides and sanguinarine is likely due to the fact that aminoglycoside enhanced membrane permeability and faciliated the penetration of sanguinarine (Figs 4 and 6). Sanguinarine was known to generate ROS upon the entry of mammalian cells (Choi *et al*., 2008). ROS production during aminoglycoside treatment was considered as one of the mechanisms for bacterial death (Kohanski *et al*., 2007; Dwyer *et al*., 2014). Deletion of IscS, the essential component for the synthesis of the Fe‐S cluster, was shown to abolish the production of ROS and thus alleviated the aminolgycoside killing (Kohanski *et al*., 2007, 2008; Dwyer *et al*., 2014). The role of ROS in aminoglycoside killing and *iscS* in ROS production is still controversal (Ezraty *et al*., 2013). Our results showed that despite sanguinarine alone at our used concentration did not produce a significant level of ROS, the combination with aminoglycoside significantly elevated the endogenous level of ROS (Fig. 6). Furthermore, the increase in intracellular ROS level in bacteria treated by the combination of sanguinarine and kanamycin required an intact Fenton reaction (Fig. 3C). Previous studies have shown that ROS leads to the oxidation of the guanine nucleotide pool, which contributes to bacterial death (Foti *et al*., 2012). In this research, the increase in intracellular ROS level correlates well with the bacterial death upon the treatment as sanguinarine lost the potentiation on aminoglycoside killing when ROS production was abolished chemically or genetically (Fig. 3A–D), suggesting the important role of ROS in sanguinarine‐mediated potentiation on aminoglycoside killing (Fig. 6).

One intriguing question is that why the potentiation of sanguinarine was only observed on aminoglycosides but not on other bactericidal antibiotics, such as β‐lactams and fluoroquinolones? Our results indicate that this observation likely attribute to the enhancement of bacterial membrane permeability by aminoglycosides. It was long known that aminoglycosides could produce mistranslated and misfolded proteins and the integration of such proteins into bacterial membrane could corrupt bacterial membrane and eventually enhanced the permeability (Davis *et al*., 1986; Davis, 1987). We reason that the treatment of bacteria with aminoglycosides enhanced bacterial membrane permeability, which in turn allows the accumulation of a higher level of sanguinarine inside bacterial. Indeed, making use of the fluorescence spectrum of sanguinarine, we demonstrated the enhanced accumulation of sanguinarine inside bacterial cells treated by aminoglycosides (Figs 4 and 6). However, this accumulation of sanguinarine was not observed inside bacteria treated with β‐lactams or fluoroquinolones. The elevated intracellular sanguinarine concentration was believed to enhance the killing activity of aminoglycoside. Therefore, aminoglycosides enhancing the uptake of sanguinarine could be the unique mechanism of the synergistic killing effect of sanguinarine and aminoglycoside.

Sanguinarine has been widely used in traditional medicine for centuries. Due to benefit from its antimicrobial and anti‐inflammation activity, sanguinarine has been included as an ingredient in toothpaste (Laster and Lobene, 1990; Godowski *et al*., 1995). However, recent studies have raised concerns about its toxicity (Singh and Sharma, 2018). While further investigation is still needed on its toxicity due to the controversies results from different experiments (Ahmad *et al*., 2000; Eversole *et al*., 2000; Chan, 2015; Zhong *et al*., 2017), we should be cautious on the direct use of sanguinarine on the human beings. The safe dose of sanguinarine should be carefully evaluated and a detailed structure and activity relationship (SAR) study should be conducted to find derivatives with low or no toxicity. Sanguinarine has been used in poultry as the feeding additive (Hassan *et al*., 2018). Efficient antibiotic therapy is still crucial for the broiler industry to treat infectious diseases (Sharma *et al*., 2016). Using aminoglycoside to treat poultry fed with sanguinarine might efficiently cure the infections. Nevertheless, the identification of sanguinarine to potentiate aminoglycoside killing with the unique mechanism provides an intriguing approach to revive a broad range of aminoglycoside antibiotics for treatment on drug‐resistant bacterial infection.

## Experimental procedures

### Bacterial strains and growth conditions

The bacterial species and strains used in this study are listed in Table S1. Luria‐Bertani (LB) broth was used for bacterial culture and mutant construction. M9 minimum medium (8.56 mM NaCl, 18.69 mM NH_4_Cl, 22.04 mM KH_2_PO_4_, 47.76 mM Na_2_HPO_4_, 4 mM MgSO_4_, 0.2 mM CaCl_2_, 3 μM thiamine, 0.1% casamino acid and 0.8% glucose) was used for MIC determination, library screening, checkerboard assay, killing assay and biofilm killing assay.

### Plasmid and bacterial strains construction

In‐frame deletion mutant Δ*iscS* was constructed based on *Sac*B‐based allelic exchange with suicide plasmid pDS132 as described previously (Philippe *et al*., 2004; Zheng *et al*., 2005). *E*. *coli* MG1655 was used as the parental strain. Briefly, primer sets iscs_up_for/rev and iscS_down_for/rev (Table S2) were used to amplify the upstream and downstream flanking sequence of *iscS*. The resultant PCR products that include a fragment containing 800 bp of the upstream of *iscS* and a fragment containing 1077 bp of the downstream of *iscS* were cloned into pDS132 plasmid and then mobilized into *E*. *coli* MFD λ pir. Double cross‐over deletion mutants were obtained and verified by PCR.

### Compound library screening

Apoptosis compound library (Selleck, L3300‐Z427549) consisting of 693 chemical compounds from Selleck Chemicals was used for this screening. Briefly, *E*. *coli* MG1655 was cultured in LB broth at 37°C overnight and then subcultured into M9 medium at a ratio of 1:100 and continued to be cultured at 37°C, 220 rpm until OD_600_ reached 0.5. Bacteria were then diluted to OD_600_ = 0.04 and 100 μl bacterial culture was then added to each well of the 96‐well plate, which contained 100 μl M9 medium with 80 μM compound and 6 mg l^−1^ kanamycin (1/2 MIC) (final concentration: compound 40 μM and kanamycin 1/4 MIC). The plates were kept at 37°C for 16 h and the bacterial growth was then examined by measure OD_600_ using a SpectraMax M5 multi‐detection microplate reader system (Molecular Devices, San Jose, California, USA). Compounds that resulted in bacterial growth with OD_600_ < 0.1 were considered as the primary hits. A similar procedure was employed for the secondary screening.

### Chemicals and antibiotics

Sanguinarine chloride (Aladdin, S1010540，Shanghai, China). l‐Glutathione reduced (Sigma, G4251, Darmstadt, Germany). Kanamycin sulfate (Thermo Fisher Scientific, J17924.06, Carlsbad, California, USA). Tobramycin sulfate salt (Sigma, T1783, Darmstadt, Germany). Gentamicin sulfate (Sigma, G1914, Darmstadt, Germany). Streptomycin sulfate (Fisher Scientific, BP910‐50, Carlsbad, California, USA). Neomycin trisulfate salt hydrate (Sigma, N1876). Amikacin disulfate salt (Sigma, A1774, Darmstadt, Germany). Ampicillin sodium salt (Fisher Scientific, BP1760, Carlsbad, California, USA). Norfloxacin (MP Biomedicals, 155949, Solon, Ohio, USA).

### MIC determination

Minimum inhibitory concentration was measured using the broth microdilution method as described previously (Andrews, 2001). Twofold serial dilution of each antibiotic was made in a 96‐well plate with 100 μl M9 medium in each well. Bacteria were cultured in M9 overnight and then subcultured into fresh M9 medium at a ratio of 1:100. Bacteria were incubated in a shaking incubator at 37°C, 220 rpm until OD_600_ reached 0.5. Bacterial culture was diluted to OD_600_ = 0.04 with M9 medium. Hundred microlitre of bacterial suspension was added to a 96‐well plate which contained the serially diluted antibiotics. The plates were then incubated at 37°C for 16 h. The OD_600_ was measured using a SpectraMax M5 multi‐detection microplate reader system. The lowest concentration that inhibits the visible growth of bacteria was defined as the MIC.

### Checkerboard assay and drug synergy analysis

Checkerboard assay was performed following previous protocol with minor modification (Orhan *et al*., 2005). Briefly, a fixed concentration of aminoglycosides was added to the 96‐well plate in which sanguinarine with twofold serial dilution by M9 medium has already been added. The final volume of the drug mixture was topped up to 100 μl and then 100 μl bacterial suspension (OD_600_ = 0.04) was added. Plates were incubated at 37°C for 16 h. OD_600_ was measured by SpectraMax M5. Drug synergy or antagonism was analysed by utilizing Combenefit software with Bliss independence criterion and the Loewe additivity model (Greco *et al*., 1992; Di Veroli *et al*., 2016).

### Bacterial killing assay

Bacteria killing assay was performed as described previously with minor modification (Ji *et al*., 2019). Briefly, bacterial cultures at the exponential phase were transferred to a 12 ml Falcon round bottom tube and incubated with indicated antibiotic or/and sanguinarine with aeration at 37°C at 220 rpm for 6 h or for indicated times in the dynamic killing assays. Sanguinarine concentration was 20 μM. The concentrations of antibiotics were used at 1× MIC (Table S3). At the indicated time point, 500 μl of bacteria was transferred into a 1.5 ml Eppendorf (EP) tube and centrifuged at 8000 rpm for 3 min. The resulting pellet was resuspended in 500 μl sterile 1× phosphate‐buffered saline (PBS). The suspension was subject to10‐fold serial dilution and the bacterial survivors were examined by a plating method.

### Biofilm killing assay

For biofilm killing assay, 50 μl of stationary phase bacteria cultured in LB broth were diluted 1:100 into 5 ml fresh LB broth in a 12 ml Falcon round bottom tube. Bacteria were cultured with 6 mm of PE50 catheters (0.58 mm * 0.96 mm) in a static incubator at 37°C for 72 h to form biofilms. The PE50 catheters were then washed with sterile 1× PBS five times to remove the loosely adherent cells. Six PE50 catheters were transferred into a 1.5 ml Eppendorf tube and were cultured in 1 ml M9 medium with tested antibiotics or/and sanguinarine at 37°C at 220 rmp. After 6 h treatment (24 h for *P*. *aeruginosa* PAO1 and *K*. *pneumonia* ATCC 13833), catheters were removed and put into a fresh 1.5 ml Eppendorf tube containing 1 ml of sterile 1× PBS and were then sonicated for 5 min twice to release the biofilm cells. The solution was subject to 10‐fold serial dilution, followed by plating to examine the bacterial survivors. For confocal microscope observation, *P*. *aeruginosa* PAO1 was inoculated in a 6‐well plate in which each well contained 4 ml LB broth and glass cover slides at the bottom. Bacteria were cultured at 37°C for 72 h to form biofilms. The medium was gently removed and planktonic bacteria were removed by washing twice with 1× PBS. Adherent biofilm cells were incubated in 3 ml M9 medium with gentamicin and sanguinarine alone or their combinations at 37°C for 6 h. SYTO 9 at 5 μM (Invitrogen, S34854， Carlsbad, California, USA) and PI at 30 μM (Invitrogen, P3566, Carlsbad, California, USA) were applied to stain the bacteria for 20 min. Images were taken by Carl Zeiss LSM710 Confocal.

### ROS and Sanguinarine penetration determination

Bacteria were treated following the same procedure as described in the killing assay. At 30 min post‐treatment, 1 ml of bacterial culture was transferred into a 1.5 ml EP tube and carboxy‐H2DCFDA (Invitrogen, C400) or HPF (Invitrogen, H36004) was added for staining. Both carboxy‐H2DCFDA and HPF were used at a concentration of 10 μM. Staining was performed at dark at 37°C in a shaking incubator. At 30 min post staining, bacteria were washed once with sterilized 1× PBS and were then analysed by flow cytometry (Beckman coulter CytoFLEX S, Laser excitation: 488 nm; emission detection: FL1 525/40 nm). For sanguinarine penetration measurement, at 1 h post‐treatment, bacteria were collected and washed once with fresh 1× PBS, then the fluorescence intensity was examined by flow cytometry (Beckman coulter CytoFLEX S, Laser excitation: 488 nm; emission detection: FL1 690/50 nm).

### 8‐oxo‐dG detection

For 8‐oxo‐dG examination, exponential phase *E*. *coli* MG1655 were treated with kanamycin, sanguinarine, or their combinations. At 3 h post the treatment, bacteria from each group were collected and adjusted to the same density according to OD_600_ value. A volume of 6 ml bacterial liquid was centrifuged at 5000 rpm for 5 min. Supernatants were discarded and the bacterial pellet was then resuspended in 200 μl pre‐cold sterilized 1× PBS. The bacterial resuspension was then sonicated for 2 min at 4°C with sonication program: amplitude 40, run 5 s pause 5 s. Bacterial lysis was centrifuged at 1000 rpm for 10 min. Supernatants were collected to examine 8‐oxo‐dG following the user manual of the 8‐OHdG ELISA Kit (Sangon Biotech, D751009, Shanghai, China).

### TUNEL assay

Apo‐Direct Kit (BD Bioscience, 556381) was used to perform the TUNEL assay as described previously (Dwyer *et al*., 2012; Dwyer and Winkler, 2013). Briefly, at 3 h post the indicated treatment, 1 × 10^7^ bacterial cells were collected and washed by 1× PBS once. The bacterial pellet was then resuspended in 1 ml of cold 4% (w/v) paraformaldehyde in 1× PBS solution and fixed on ice for 30 min. Cells were centrifuged, washed once with 1× PBS and resuspended in 200 μl cold 70% ethanol. The resuspension was kept on ice for 30 min for permeabilization. For staining, samples were washed twice with wash buffer (kit component) and resuspended in 50 μl of staining solution (kit component). The mixture was kept in dark at 37°C with shaking at 220 rpm for 1 h. A volume of 1 ml rinse buffer from the kit was added to stop the reaction. The buffer was then removed by centrifugation. Cells were pelleted and were then resuspended in 1× PBS for observation with a fluorescence microscope (Leica THUNDER Imager 3D assay‐DMi8 Automated Inverted Microscope with Fluorescence, FITC filter: excitation: 480/40, emission: 527/30).

### Animal infection model

Mouse chronic urinary tract infection experiments were conducted following the previous description with 6–8 weeks old female C57BL/6J mice (Allison *et al*., 2011). Biofilm was grown in PE50 catheter as described in biofilm killing assay. Mice were anesthetized by 250 mg kg^−1^ avertin treatment and then received surgical implantation in the urinary tract of 6 mm PE50 catheter grown with biofilm. At 48 h after the implantation, mice were randomly divided into four groups (*n* = 5) and then injected intraperitoneally with saline, kanamycin (3 mg kg^−1^) or/and sanguinarine (3 mg kg^−1^). Treatments were executed every 12 h and lasted for 4 days. At 24 h after the last treatment, catheters were aseptically collected to determine biofilm viability as described in the biofilm killing assay.

The mouse thigh biofilm infection experiment was conducted following the previous description with 6‐week old male C57BL/6J mice (Conlon *et al*., 2013). Mice were first rendered neutropenic by cyclophosphamide administration (Zuluaga *et al*., 2006). *E*. *coli* MG1655 of a dose of 1 × 10^6^ in 100 μl 1× PBS were injected into the left thigh of each mouse. Infection was allowed to develop for 12 h before the commencement of treatment by kanamycin, sanguinarine or their combinations. Mice were randomly divided into four groups (*n* = 6) and were treated with saline, kanamycin (3 mg kg^−1^) or/and sanguinarine (3 mg kg^−1^) once every 12 h for 2 days. At 12 h after the last therapy, mice were sacrificed by euthanasia and the thigh muscle of the left leg of the mice was removed and homogenized in 1 ml sterile 1× PBS. Homogenates were subject to 10‐fold serial dilution and 100 μl samples of each dilution were spread onto LB agar plate for the enumeration of bacterial survivors. The animal experiments were approved by the Panel on Research Ethics of University of Macau (UMARE‐018‐2019).

### Statistical analysis

GraphPad Prism was utilized to perform statistical analysis. All statistical comparisons were performed using unpaired *t*‐tests otherwise specifically stated. *P*‐value < 0.05 was considered significant (∗*P* < 0.05, ∗∗*P* < 0.01, ∗∗∗*P* < 0.001).

## Conflict of interest

None declared.

## Supporting information


**Table S1.** Strains list.
**Table S2.** Primers for *iscS* deletion.
**Table S3.** MIC of strains (mg l^−1^).

**Figure S1.** Sanguinarine potentiates the antibacterial activity of aminoglycosides in gram‐negative bacteria only. (A‐C) Checkerboard plots (top) for antibacterial activity of sanguinarine in combination with indicated antibiotics on *E. coli* MG1655 (A and B) and *S. aureus* 25904 (C), and the corresponding synergistic effect analyzed by Bliss independence criterion was shown (bottom). SAN, sanguinarine; Str, streptomycin; Neo, neomycin; Ami, amikacin; Amp, ampicillin; Norf, norfloxacin.
**Figure S2.** Synergy determination by Loewe additive model on sanguinarine with various of antibiotics in *E. coli* (A), *K. pneumonia* (B) and *S. aureus* (C). The synergy and antagonism was analyzed by Loewe additivity model. SAN, sanguinarine; Kan, kanamycin; Tob, tobramycin; Gen, gentamicin; Str, streptomycin; Neo, neomycin; Ami, amikacin; Amp, ampicillin; Norf, norfloxacin.
**Figure S3.** Sanguinarine potentiates bacterial killing activity on different aminoglycosides. (A) Sanguinarine enhanced the bacterial killing activity of different aminoglycosides. *E. coli* MG1655 was treated by 1×MIC of indicated aminoglycosides, 7.5 mg/L sanguinarine or their combinations for 6 h and the bacterial survivors were examined. ^‘#’^ represents that the CFUs are under the limit of detection. Data are presented as mean ± SD. Statistical analysis was performed using un‐paired t test. * p<0.05; ** p<0.01; *** p<0.001. (B) and (C) Sanguinarine potentiates the bacterial killing activity of kanamycin on A. baumannii ATCC 17978 (B) and P. aeruginosa PAO1 (C). 1×MIC aminoglycosides, 15 mg/L sanguinarine or their combinations was used for the experiments. The killing assay was performed at least three times in triplicate. Data are representative of the three independent experiments. The results represent means ± SD (n=3). Ctrl, control; SAN, sanguinarine; Tob, tobramycin; Gen, Gentamicin; Str, Streptomycin; Kan, kanamycin.
**Figure s4.** The elevation of ROS in bacteria treated by the combination of sanguinarine and kanamycin. (A) The production of ROS was examined in K. pneumoniae ATCC 13833 after 1 h treatment with 1×MIC of kanamycin, 20 μM sanguinarine, or their combinations with carboxy‐H2DCFDA (right) or with HPF (left). (B) ROS in *E. coli* MG1655 after treatment with 1×MIC of kanamycin with or without 7.5 mg/L sanguinarine in the presence or absence of 20 mM GSH were detected by HPF. (C) ROS in *E. coli* MG1655 wildtype and ΔiscS mutant after 1 h treatment with 1×MIC of kanamycin, 7.5 mg/L sanguinarine or their combinations. Ctrl, control; SAN, sanguinarine; Kan, kanamycin; GSH, glutathione.
