# Supplementary material for: Sanguinarine synergistically potentiates aminoglycoside‐mediated bacterial killing
Source: Microb Biotechnol. 2022 Mar 23;15(7):2055–70. doi: 10.1111/1751-7915.14017 (PMC9249330; doi:10.1111/1751-7915.14017)
Supplement: Supplementary file 1 — Table S1. Strains list. Table S2. Primers for iscS deletion. Table S3. MIC of strains (mg l−1). [file MBT2-15-2055-s002.docx]

**Supporting Tables**

| Supporting Table1: Strains list | | | | |  |
| --- | --- | --- | --- | --- | --- |
| Strains | Application | | | |  |
| *E. coli* MG1655 | Compound library screening, checkerboard assay, killing assay, biofilm killing assay, animal infection, mutant construction | | | | |
| *K. pneumoniae* ATCC 13833 | Checkerboard assay, killing assay, biofilm killing assay | | | | |
| *S. aureus* ATCC 25904 | Checkerboard assay | |  |  |  |
| *A. baumannii* ATCC 17978 | Killing assay |  |  |  |  |
| *P. aeruginosa* PAO1 | Killing assay, biofilm killing assay | | |  |  |
| UPEC UTI01 | Biofilm killing assay, animal infection | | |  |  |
| *E. coli* MFD λ pir | Mutant construction | |  |  |  |
| *E. coli* DH5α λ pir | Mutant construction | |  |  |  |

| Supporting Table 2: Primers for *iscS* deletion | |  |
| --- | --- | --- |
| Primer | Secquence(5'-3') |  |
| iscs_up_for | aaaaggatcgatcctctagacaaaccccacgcgcaggc | |
| iscs_up_rev | tgtacggagtttatagagcatcggtatcggaatcagg | |
| iscs_down_for | tgtacggagtttatagagcatcggtatcggaatcagga | |
| iscs_down_rev | cgcatgcggtacctctagaagaataaaggtaacaaggcc | |
| ide_iscs_up_for | gcgcctgggtatcgagttgata |  |
| ide_iscs_up_rev | gaattcagaatcaggccggagtgct |  |
| ide_iscs_down_for | ctcgtcgttgttgtcaaagg |  |
| ide_iscs_down_rev | aatatcctgcgcgggattct |  |

| Supporting Table 3: MIC of strains (mg/L) | | | | | | |
| --- | --- | --- | --- | --- | --- | --- |
|  | Kan | Gen | Tob | Str | Amp | Norf |
| *E. coli* K-12 MG1655 | 12 | 3 | 3 | 12 | 6 | 0.25 |
| *K. pneumoniae* ATCC 13833 | 6 | 3 | 3 | -- | -- | -- |
| *A. baumannii* ATCC 17978 | 6 | -- | -- | -- | -- | -- |
| *P. aeruginosa* PAO1 | -- | 5 | -- | -- | -- | -- |
| UPEC UTI01 | 10 | -- | -- | -- | -- | -- |
