# Supplementary material for: Sanguinarine synergistically potentiates aminoglycoside‐mediated bacterial killing
Source: Microb Biotechnol. 2022 Mar 23;15(7):2055–70. doi: 10.1111/1751-7915.14017 (PMC9249330; doi:10.1111/1751-7915.14017)
Supplement: Supplementary file 2 — Figure S1. Sanguinarine potentiates the antibacterial activity of aminoglycosides in gram‐negative bacteria only. (A‐C) Checkerboard plots (top) for antibacterial activity of sanguinarine in combination with indicated antibiotics on E. coli MG1655 (A and B) and S. aureus 25904 (C), and the corresponding synergistic effect analyzed by Bliss independence criterion was shown (bottom). SAN, sanguinarine; Str, streptomycin; Neo, neomycin; Ami, amikacin; Amp, ampicillin; Norf, norfloxacin. Figure S2. Synergy determination by Loewe additive model on sanguinarine with various of antibiotics in E. coli (A), K. pneumonia (B) and S. aureus (C). The synergy and antagonism was analyzed by Loewe additivity model. SAN, sanguinarine; Kan, kanamycin; Tob, tobramycin; Gen, gentamicin; Str, streptomycin; Neo, neomycin; Ami, amikacin; Amp, ampicillin; Norf, norfloxacin. Figure S3. Sanguinarine potentiates bacterial killing activity on different aminoglycosides. (A) Sanguinarine enhanced the bacterial killing activity of different aminoglycosides. E. coli MG1655 was treated by 1×MIC of indicated aminoglycosides, 7.5 mg/L sanguinarine or their combinations for 6 h and the bacterial survivors were examined. ‘#’ represents that the CFUs are under the limit of detection. Data are presented as mean ± SD. Statistical analysis was performed using un‐paired t test. * p<0.05; ** p<0.01; *** p<0.001. (B) and (C) Sanguinarine potentiates the bacterial killing activity of kanamycin on A. baumannii ATCC 17978 (B) and P. aeruginosa PAO1 (C). 1×MIC aminoglycosides, 15 mg/L sanguinarine or their combinations was used for the experiments. The killing assay was performed at least three times in triplicate. Data are representative of the three independent experiments. The results represent means ± SD (n=3). Ctrl, control; SAN, sanguinarine; Tob, tobramycin; Gen, Gentamicin; Str, Streptomycin; Kan, kanamycin. Figure s4. The elevation of ROS in bacteria treated by the combination of sanguinarine and kanamyc [file MBT2-15-2055-s001.docx]

**Sanguinarine synergistically potentiates aminoglycoside - mediated bacterial killing**

Chang Lu^1^, Nian Zhang^1^, Sihoi Kou^1^, Liangliang Gao^1^, Bo Peng^2,3^, Yunlu Dai^1,4 *^ and Jun Zheng^1,4 *^

**Affiliations:**

^1^ Faculty of Health Sciences, University of Macau, Macau SAR, China;

^2^ School of Life Sciences, Sun Yat-sen University, Guangzhou 510006, China;

^3^ Laboratory for Marine Biology and Biotechnology, Qingdao National Laboratory for Marine Science and Technology, Qingdao 266071, China;

^4^ Institute of Translational Medicine, University of Macau, Macau SAR, China.

^*^ Correspondence should be addressed to:

Jun Zheng, E-mail: [zhjunyy@gmail.com](mailto:junzheng@um.edu.mo); Tel: +853 8822 4509; Fax: +853 8822 2314; or Yunlu Dai, E-mail: [yldai@um.edu.mo](mailto:yldai@um.edu.mo); Tel: +853 8822 4881; Fax: +853 8822 2314.

**Running title:** Sanguinarine Potentiates Killing by Aminoglycoside

**Keywords:** Antibiotic resistance, bacterial persisters, sanguinarine, aminoglycoside, reactive oxygen species

**Supporting Information**

**Supporting Figures**

**
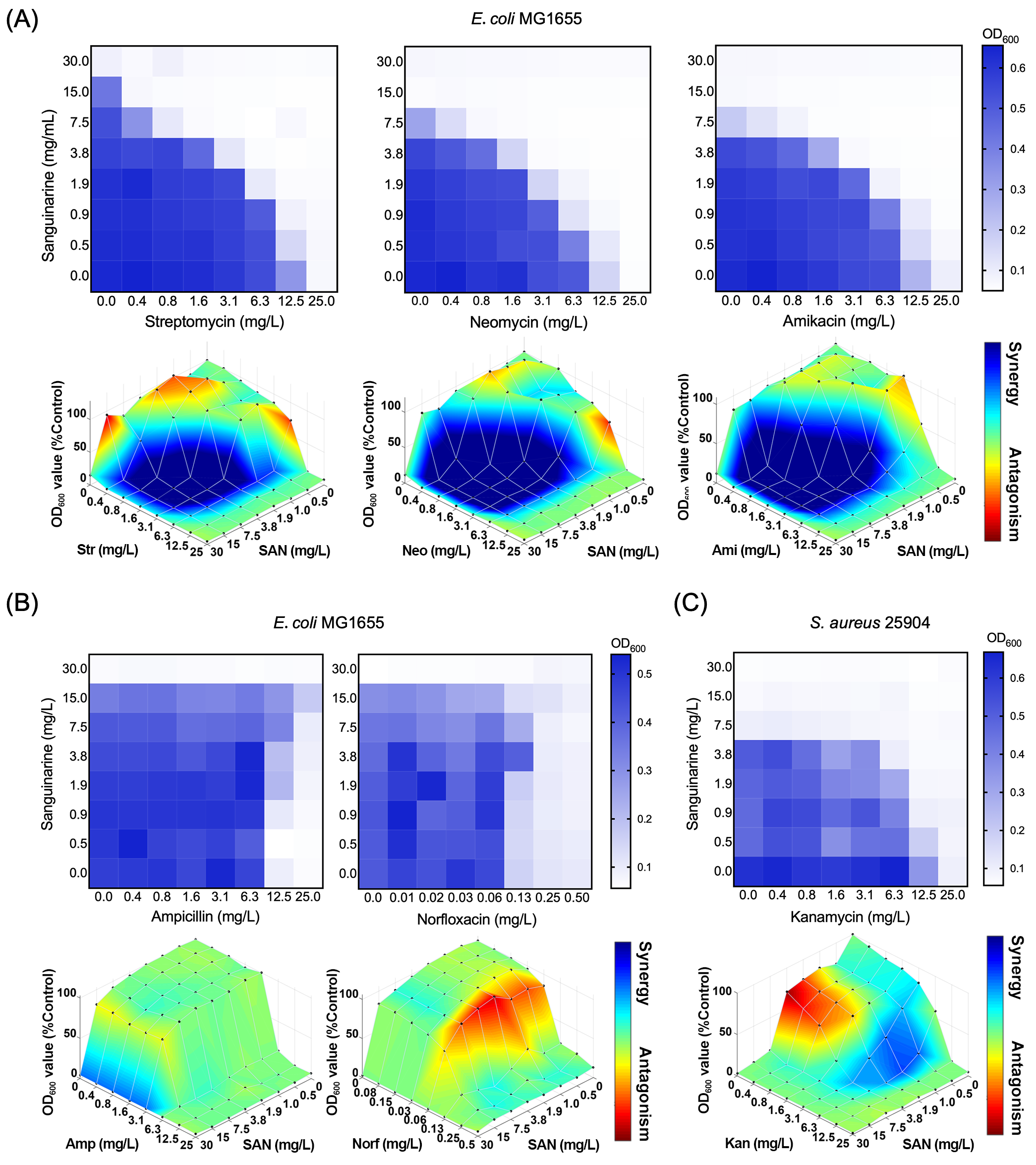
**

**Figure S1. Sanguinarine potentiates the antibacterial activity of aminoglycosides in gram-negative bacteria only. (A-C)** Checkerboard plots (top) for antibacterial activity of sanguinarine in combination with indicated antibiotics on *E. coli* MG1655 (A and B) and *S. aureus* 25904 (C), and the corresponding synergistic effect analyzed by Bliss independence criterion was shown (bottom). SAN, sanguinarine; Str, streptomycin; Neo, neomycin; Ami, amikacin; Amp, ampicillin; Norf, norfloxacin.

**
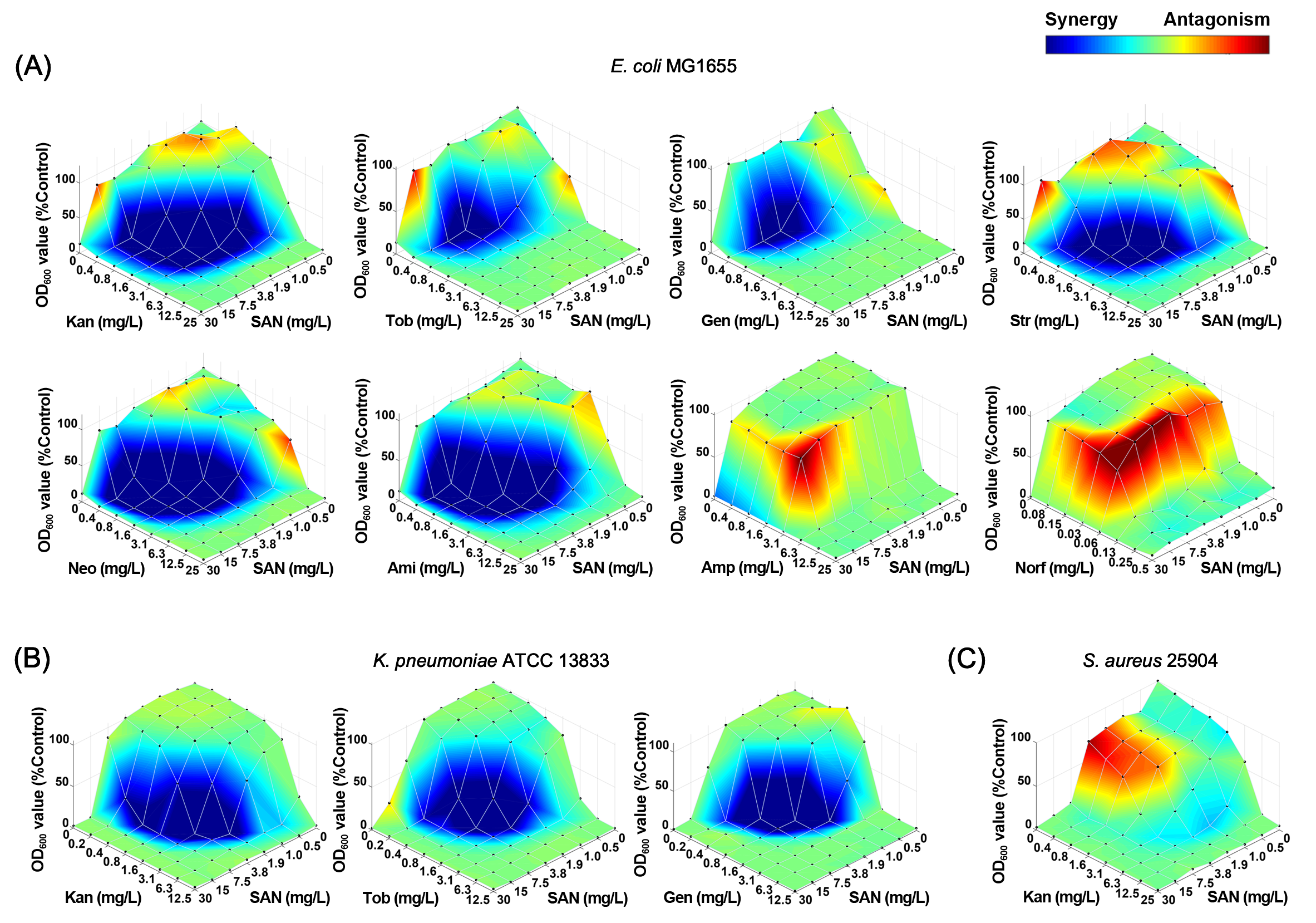
**

**Figure S2. Synergy determination by Loewe additive model on sanguinarine with various of antibiotics in *E. coli* (A), *K. pneumonia* (B) and *S. aureus* (C).** The synergy and antagonism was analyzed by Loewe additivity model. SAN, sanguinarine; Kan, kanamycin; Tob, tobramycin; Gen, gentamicin; Str, streptomycin; Neo, neomycin; Ami, amikacin; Amp, ampicillin; Norf, norfloxacin.

**
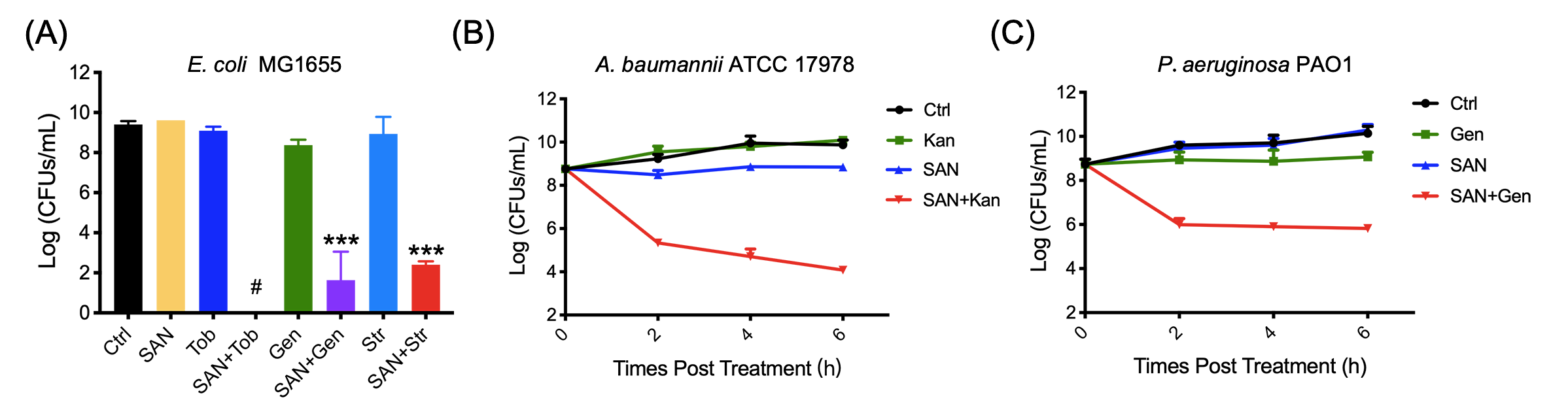
**

**Figure S3. Sanguinarine potentiates bacterial killing activity on different aminoglycosides**. **(A)**  Sanguinarine enhanced the bacterial killing activity of different aminoglycosides. *E. coli* MG1655 was treated by 1×MIC of indicated aminoglycosides, 7.5 mg/L sanguinarine or their combinations for 6 h and the bacterial survivors were examined. ‘^#^’ represents that the CFUs are under the limit of detection. Data are presented as mean ± SD. Statistical analysis was performed using un-paired *t* test. * p<0.05; ** p<0.01; *** p<0.001. **(B)** and **(C)** Sanguinarine potentiates the bacterial killing activity of kanamycin on *A. baumannii* ATCC 17978 (B) and *P. aeruginosa* PAO1 (C). 1×MIC aminoglycosides, 15 mg/L sanguinarine or their combinations was used for the experiments. The killing assay was performed at least three times in triplicate. Data are representative of the three independent experiments. The results represent means ± SD (n=3). Ctrl, control; SAN, sanguinarine; Tob, tobramycin; Gen, Gentamicin; Str, Streptomycin; Kan, kanamycin.

**
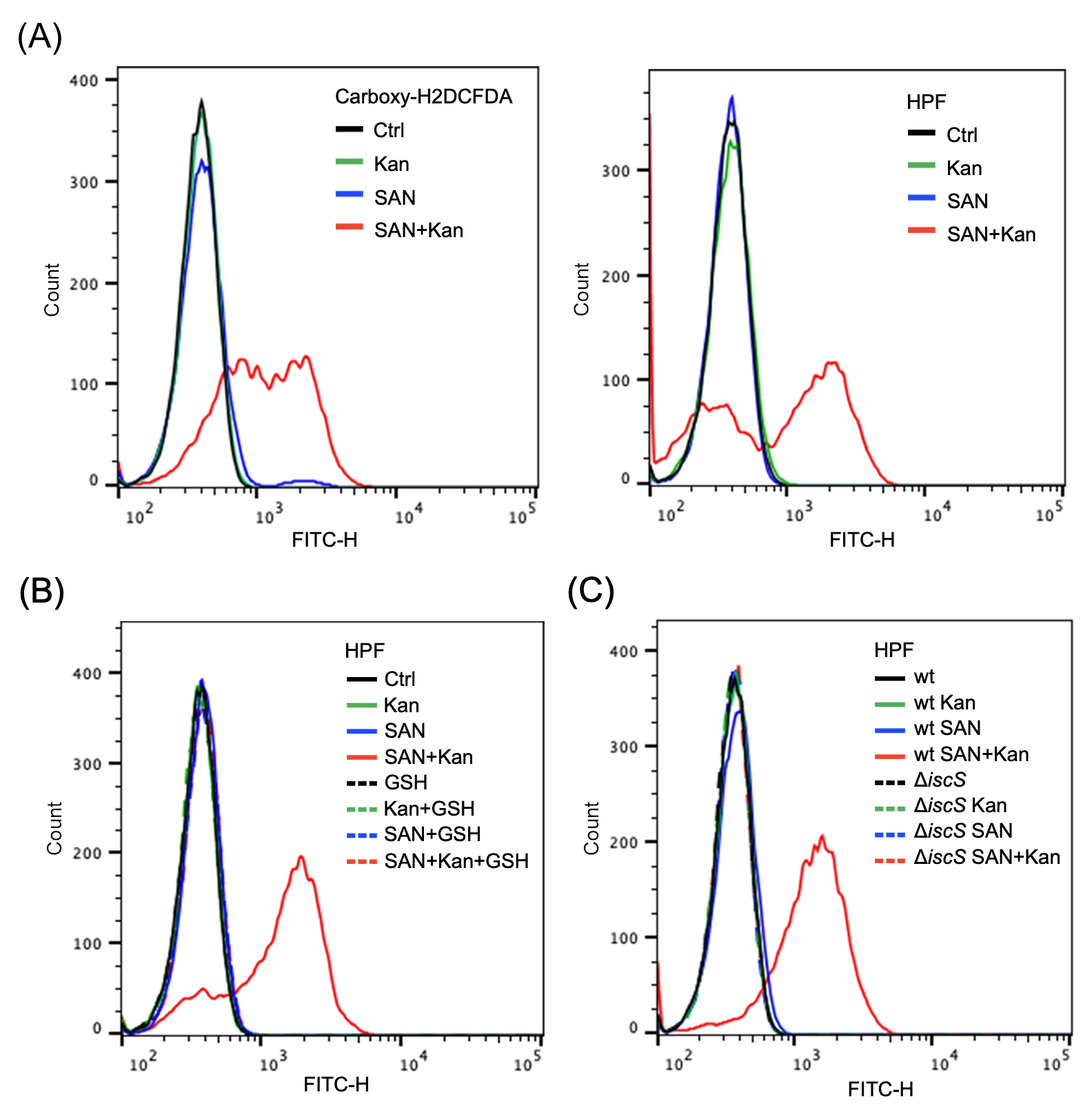
**

**Figure S4. The elevation of ROS in bacteria treated by the combination of sanguinarine and kanamycin.** **(A)** The production of ROS was examined in *K. pneumoniae* ATCC 13833 after 1 h treatment with 1×MIC of kanamycin, 20 μM sanguinarine, or their combinations with carboxy-H2DCFDA (right) or with HPF (left). **(B)** ROS in *E. coli* MG1655 after treatment with 1×MIC of kanamycin with or without 7.5 mg/L sanguinarine in the presence or absence of 20 mM GSH were detected by HPF. **(C)** ROS in *E. coli* MG1655 wildtype and Δ*iscS* mutant after 1 h treatment with 1×MIC of kanamycin, 7.5 mg/L sanguinarine or their combinations. Ctrl, control; SAN, sanguinarine; Kan, kanamycin; GSH, glutathione.
